# Ultrasensitive and low detection limit of acetone gas sensor based on ZnO/SnO_2_ thick films

**DOI:** 10.1039/d0ra06406h

**Published:** 2020-09-30

**Authors:** Yanping Chen, Yue Cao

**Affiliations:** School of Science, Shandong Jianzhu University Jinan 250101 China yanping_c@sdjzu.edu.cn; School of Physics, Shandong University Jinan 250100 China

## Abstract

In this study, we synthesized ZnO/SnO_2_ hybrid sensing nanostructures by a sol–gel method. The structures, composition and morphologies of the synthesized products were thoroughly studied by X-ray diffraction (XRD), field-emission electron scanning microscopy (FESEM) and transmission electron microscopy (TEM). After the gas sensing test, we found that the sensing performance of the ZnO/SnO_2_ composite is improved obviously compared with that of single components ZnO and SnO_2_. The response to 0.5 ppm acetone reaches 3.36, almost twice that of pure ZnO and SnO_2_. Meanwhile, the detection limit can be reduced to the ppb level. The enhanced acetone sensing performance was mainly attributed to the formation of n–n heterojunctions and the synergistic effect of ZnO and SnO_2_.

## Abstract 2

XRD patterns of ZnO/SnO_2_ powders.

## Graphical abstract

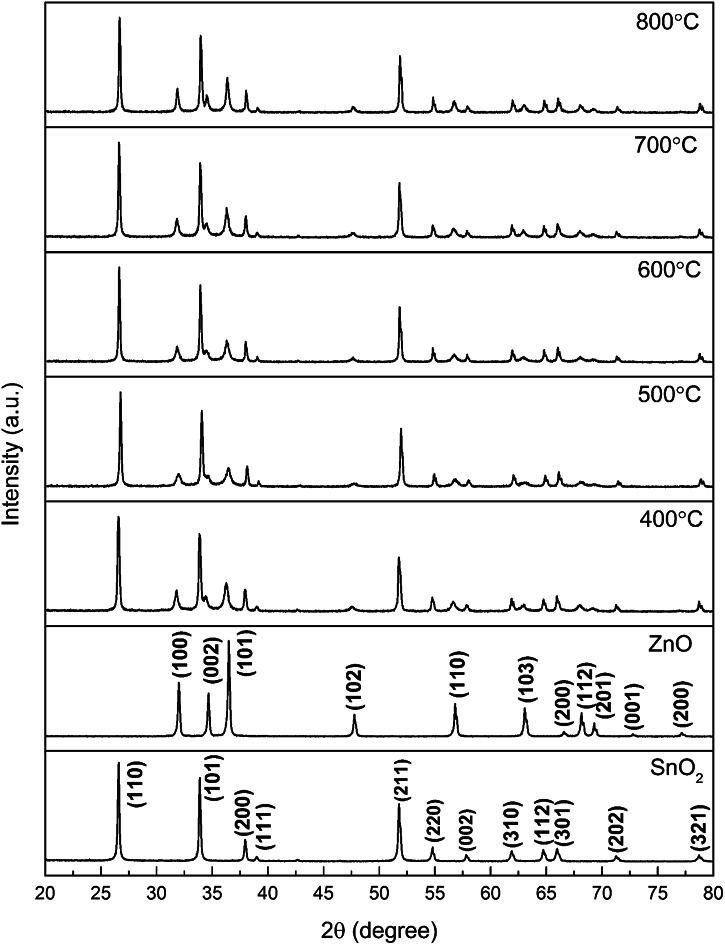

## Introduction

Acetone (C_3_H_6_O), a volatile chemical reagent in industry, has been widely used in the fields of industries, laboratories, pharmaceuticals and so on.^1^ However, acetone is harmful to human health, and may cause irritation to the throat, nose and eyes. Moreover, acetone is flammable and explosive. Recently, acetone has been widely accepted as a crucial index for noninvasive diagnosis of diabetes, since the concentration of acetone exhaled by diabetic patients (>1.8 ppm) is higher than that of healthy people (<0.9 ppm).^2^ Therefore, for the safety and health of human beings, it is of great importance to develop acetone sensing materials with high sensitivity and selectivity, high performance.

Various acetone-sensing material based on oxide semiconductors have been reported, because of their advantages of miniaturized dimensions, low cost, easy fabrication, and good reversibility. Among the many acetone gas sensing materials, ZnO and SnO_2_ are n-type semiconductors, which are one of the few materials that have been successfully commercialized for gas sensing applications.^3–5^ However, there are still some drawbacks, such as high working temperatures, low sensitivity and poor selectivity, which hinder its practical application as a high-performance gas sensor. Various strategies have been reported to improve the sensing performance of metal oxide semiconductors. Among them, engineering of heterostructure nanocomposites with other sensing units have been proved to be one of the most effective methods to enhance gas-sensing performance. Such as, Liu *et al.* synthesized heterostructure ZnO/SnO_2_ with hollow nanostructure exhibiting high response to ethanol have been reported.^6^ Z. Anajafi *et al.* reported SmFeO_3_/ZnO nanocomposite to detect acetone at low concentration.^7^ P. Pascariu *et al.* have prepared NiO doped SnO_2_ to detect humidity.^8^ Recently, H. Kim *et al.* have reported Co_3_O_4_ nanoparticle-attached SnO_2_ nanowires sensing property to acetone.^9^ Several studies have reported the sensing of Zn_2_SnO_4_ and Zn_2_SnO_4_ show excellent sensing properties to acetone.^10,11^ We wonder the acetone sensing of ZnO/SnO_2_ composites when the mole ratio of (Zn^2+^)/(Sn^4+^) is 2 : 1. On the other hand, few studies on the gas sensitivity of ZnO/SnO_2_ composites to acetone have been reported. Therefore, ZnO/SnO_2_ composites were synthesized by sol–gel method and post-annealing process to investigate the acetone sensing performance at low concentration. As expected, the ZnO/SnO_2_ composites with good dispersion showed great enhancement and lower detection limit for acetone than the single components of ZnO and SnO_2_. The sensing mechanisms of the composite was also discussed.

## Experimental

The ZnO/SnO_2_ nanoparticles were synthesized by sol–gel process. In a typical process, the synthesis procedure was as follows: SnO_2_ were first completely dissolved in moderate amounts of nitric acid solution. Zn(NO_3_)_2_·6H_2_O with mole ratio (Zn^2+^)/(Sn^4+^) = 2 : 1 were add in the above solution under continuous stirring (at 80 °C). After several minutes of stirring, citric acid was added to the solution with the mole ratio of Zn and Sn ions to equivalent mole of citric acid was 1 : 1.5. Then, some polyethylene glycol (PEG; molecular weight over 20 000) was added the solution. The solution was well stirred for several hours until the sol was formed. The sol was dried and well mill to be fine powders. Finally, the precursors were calcined in air at 400–800 °C for 4 h to obtain the composites. The pure SnO_2_ and ZnO was obtained after annealed at 600 °C for 4 h of the purchased analytical reagents. The morphologies and microstructures of the synthesized ZnO, SnO_2_ and ZnO/SnO_2_ nanoparticles were analyzed by X-ray diffraction (XRD, Cu-Kα radiation), field emission scanning electron microscope (FE-SEM), high-resolution transmission electron microscopy (HRTEM) and X-ray photoelectron spectroscopy (XPS).

The paste prepared from a mixture of ZnO/SnO_2_ with deionized water is coated on ceramic tubes to fabricate inside-heated gas sensors. To improve their stability and repeatability, the sensors were calcined in air at 240 °C for 48 h before tested. The gas-sensing properties were measured with static state gas distribution in a chamber. For n-type semiconductor, the sensitivity is defined as *S* = *R*_a_/*R*_g_ (n-type semiconductor), where *R*_a_ and *R*_g_ represent the resistances of the ZnO/SnO_2_ sensor in air and in detected gases, respectively. The room temperature relative humidity of air was about 25% RH.

## Results and discussion

**Fig. 1:**
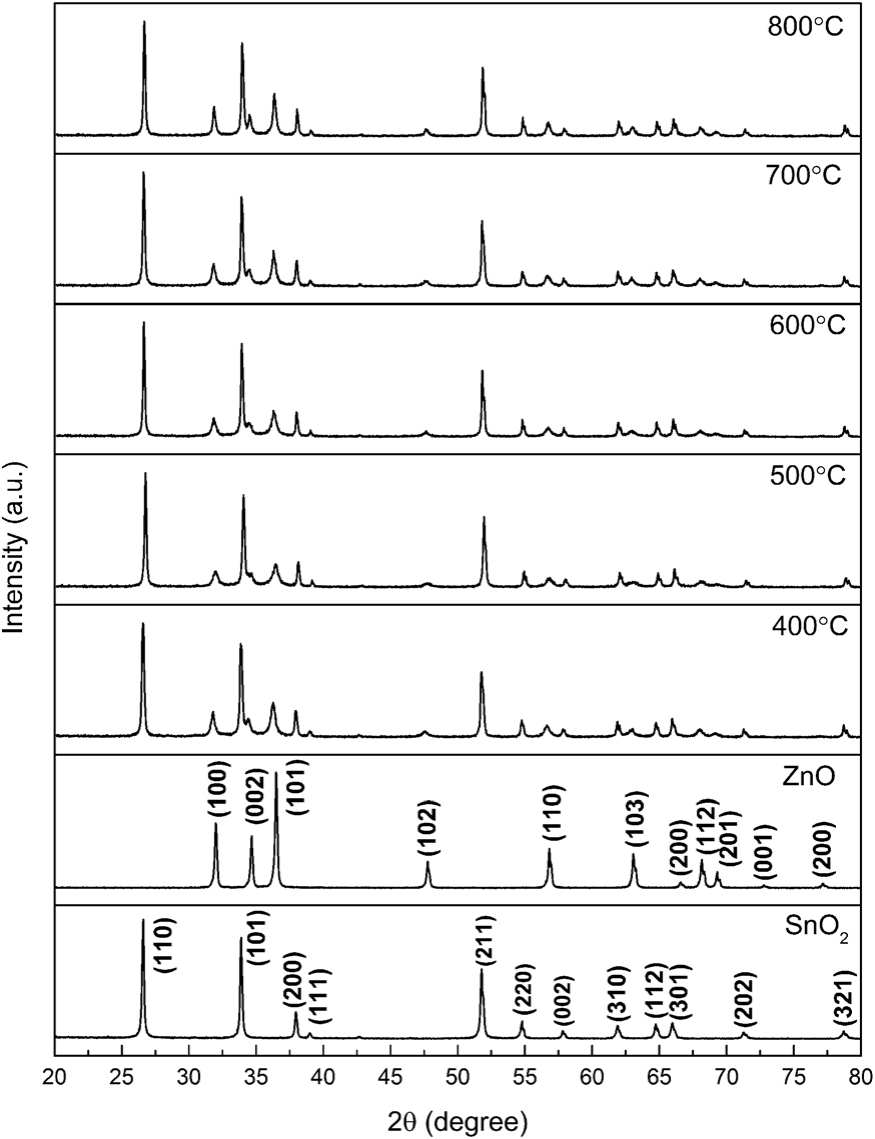
XRD patterns of ZnO/SnO_2_ powders.

**Fig. 2:**
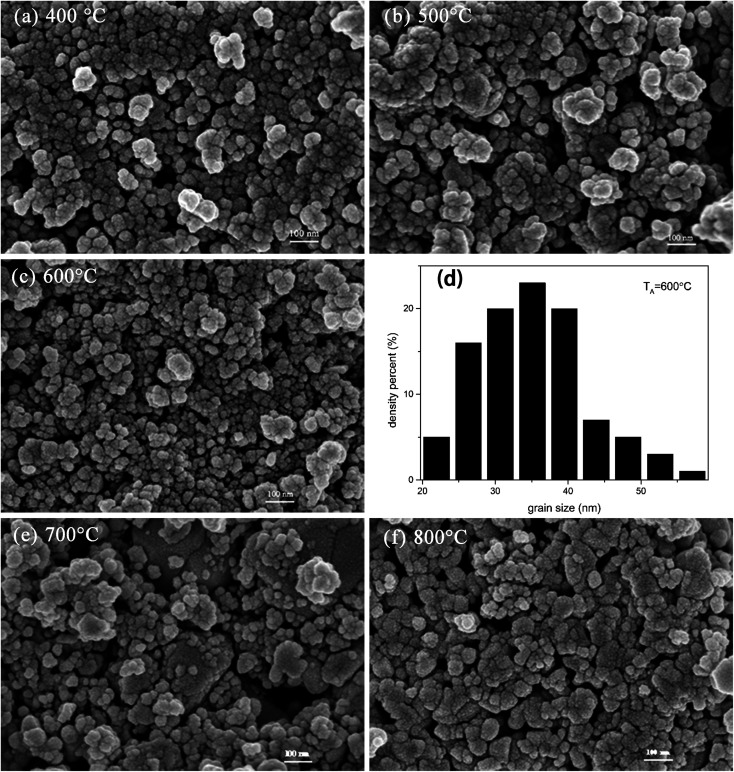
SEM images of ZnO/SnO_2_ sintered at: (a) 400 °C; (b) 500 °C; (c) 600 °C; (e) 700 °C; (f) 800 °C; (d) density percent *versus* ZnO/SnO_2_ (*T*_A_ = 600 °C) grain size.

To provide crystallinity and phase information, X-ray diffraction (XRD) experiments were performed on all the prepared nanostructured materials. Fig. 1 provides the diffraction peaks of the mixed crystal oxide phase of ZnO and SnO_2_. We know from the figure that the lattice constants are *a* = 3.249 Å, *c* = 5.206 Å, and all the diffraction peaks for pure ZnO with hexagonal wurtzite structure agree with those of JCPDS card no. 36-1451.^12^ On the indexing symbol, except that the peaks belong to pure ZnO, all the remaining diffraction peaks could be indexed to SnO_2_ (JCPDS file no. 41-1445)^13^ with rutile structure. No other diffraction peaks of impurities such as ZnSnO_*x*_ could be identified in the patterns, indicating high purity of the ZnO/SnO_2_ composites. From the XRD patterns, it can be demonstrated that ZnO and SnO_2_ phases coexisted in the products, which provide a possibility of forming n–n heterojunctions on the interface between ZnO and SnO_2_. Fig. 2 presents SEM images for ZnO/SnO_2_ composites annealing at different temperatures. As can be seen, the ZnO/SnO_2_ composites are agglomerated. Fig. 2(d) shows the density percent *versus* ZnO/SnO_2_ (*T*_A_ = 600 °C) grain size. The nanospheres are composed of particles with mean sizes of 35 nm by calculating about two hundred particles from the FE-SEM in Fig. 2(c).

**Fig. 3:**
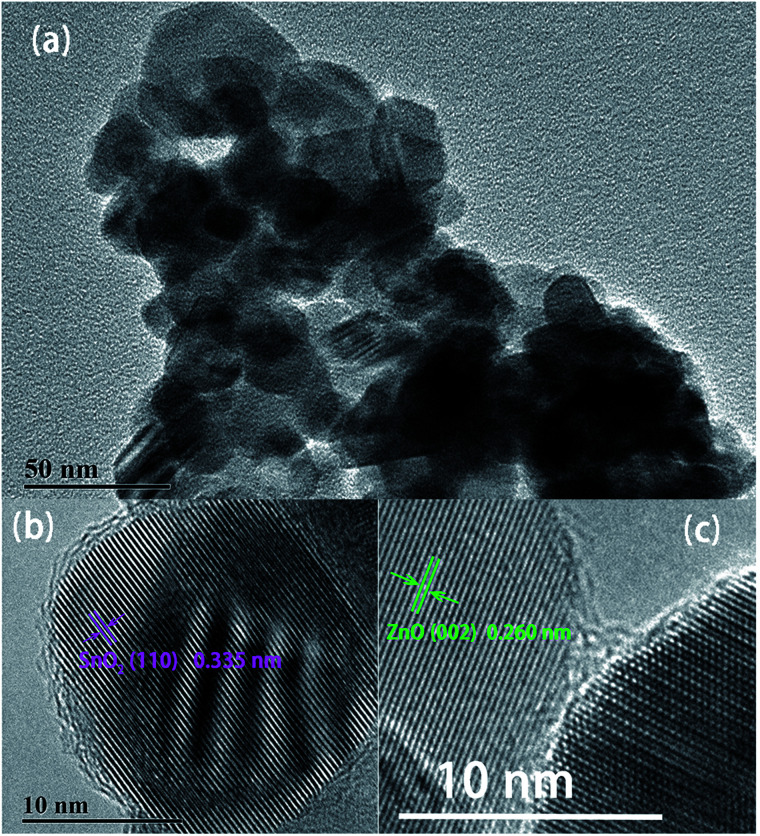
TEM images of ZnO/SnO_2_ composite sintered at 600 °C: (a) low-magnification image; (b and c) high-magnification image showing lattice fringes.

ZnO/SnO_2_ composites annealed at 600 °C was removed from all samples to further investigate the microstructure of the composite. Low-magnification TEM image of the composites is displayed in Fig. 3(a). The average particle size is in good agreement with the FESEM results in Fig. 2(c). The high-resolution TEM (HRTEM) in Fig. 3(b) and (c), reveal the lattice fringes of 0.335 nm and 0.260 nm, corresponding to the SnO_2_ (110) plane and ZnO (002) plane. The results confirmed that both the ZnO and SnO_2_ nanoparticles of the composites are single crystalline structure.

**Fig. 4:**
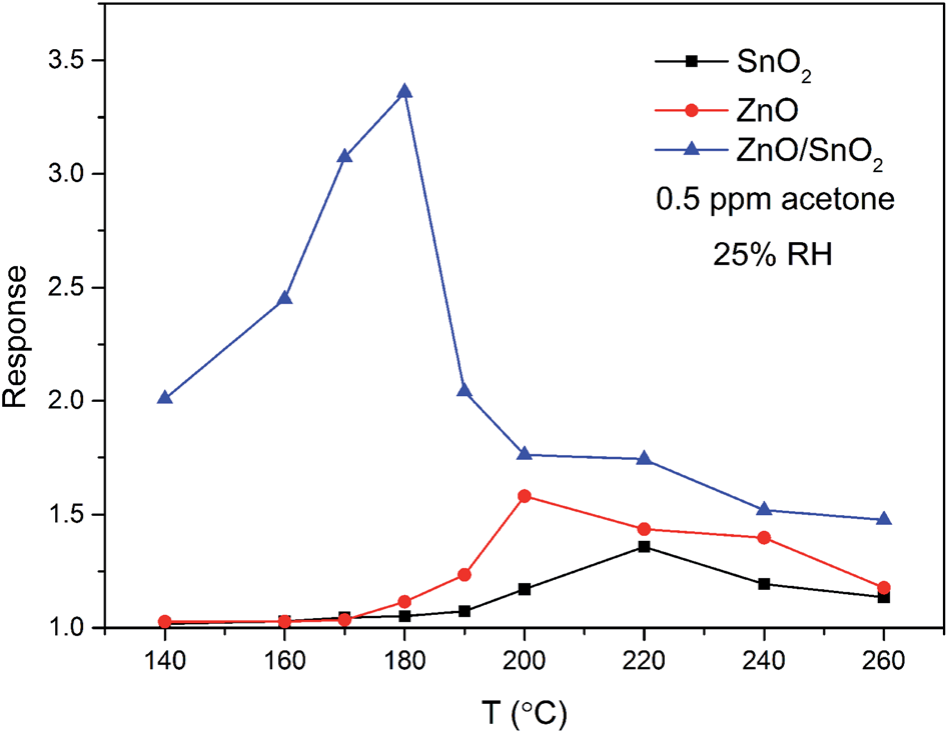
Temperature dependence of the sensitivity to 0.5 ppm acetone gas for samples ZnO, SnO_2_ and ZnO/SnO_2_.

In order to investigate the effects of combination of ZnO and SnO_2_ on gas sensitive property, sensing devices were fabricated with pure ZnO, SnO_2_ and ZnO/SnO_2_ composites. The pure ZnO, SnO_2_ and ZnO/SnO_2_ composites were annealed at 600 °C. Generally, the gas-sensing properties of semiconducting sensors are closely related to the operating temperature. Therefore, we first perform the gas sensing experiments at different working temperatures to obtain the optimum operating temperature. Fig. 4 manifests the effect of operating temperature from 140–260 °C on the sensitivities of pure ZnO, pure SnO_2_ and ZnO/SnO_2_ sensors to 0.5 ppm acetone gas. The sensitivity of all thick films increases first and then decrease with the increase of operating temperature, which is related to the different balance between adsorption and desorption of target gas on the surface of sensing film. At a lower working temperature, the insufficient thermal energy is not enough to overcome the activation energy of interface reaction, resulting in a very small response values.^14–17^ With the increase of working temperature, the responses of the sensors reach the maximum values, because there is enough thermal energy to overcome the higher barrier. When temperature exceeds the optimum working temperature, the adsorbed gas molecules may escape from the surface, resulting the gas responses decrease. The maximum responses of pure ZnO, pure SnO_2_ and ZnO/SnO_2_ sensors are attained at 190 °C (1.58), 200 °C (1.36) and 180 °C (3.36). Compared with that of ZnO and SnO_2_ samples, the response value of ZnO/SnO_2_ sensor is about 2 times larger. Besides, the optimum operating temperature of ZnO/SnO_2_ sensor shifts toward the lower temperature side. In the following investigate, we chose 180 °C as the optimum working temperature of the prepared ZnO/SnO_2_ sensing material.

**Fig. 5:**
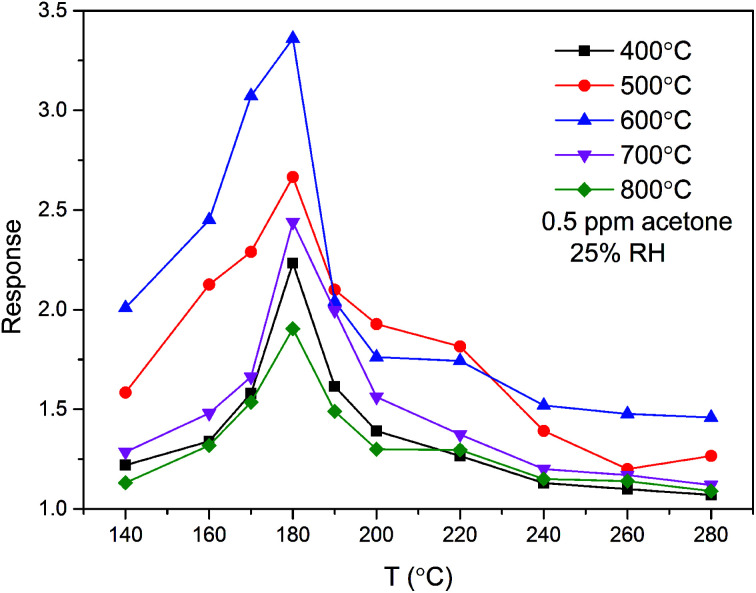
Responses of the sensors based on ZnO/SnO_2_ annealed at different temperatures as a function of operating temperature to 0.5 ppm acetone.

The annealing temperature is of vital importance for the sensing response of a gas sensor. To determine the optimal annealing temperature, the relationships between the annealing temperature and gas sensing response of the ZnO/SnO_2_ sensor for 0.5 ppm acetone were tested, and the results are shown in Fig. 5. From Fig. 5, we know that the response of ZnO/SnO_2_ annealing at 600 °C is much higher than that annealing at other temperatures. The results were consistent with the SEM image in Fig. 2. The ZnO/SnO_2_ nanoparticles annealed at 600 °C were more loosely stacked and formed a large mesoporous structure. The relatively loose mesoporous structure provides more active sites for gas molecules, thus improving its acetone gas sensing performance. At the optimal operating temperature of 180 °C, the sensors based on ZnO/SnO_2_ nanocomposites (annealed at 400 °C, 500 °C, 600 °C, 700 °C and 800 °C) present their best sensing response (2.23, 2.66, 3.36, 2.43 and 1.90, respectively) to 0.5 ppm acetone. These results clearly verify that 600 °C is the optimal annealing temperature.

**Fig. 6:**
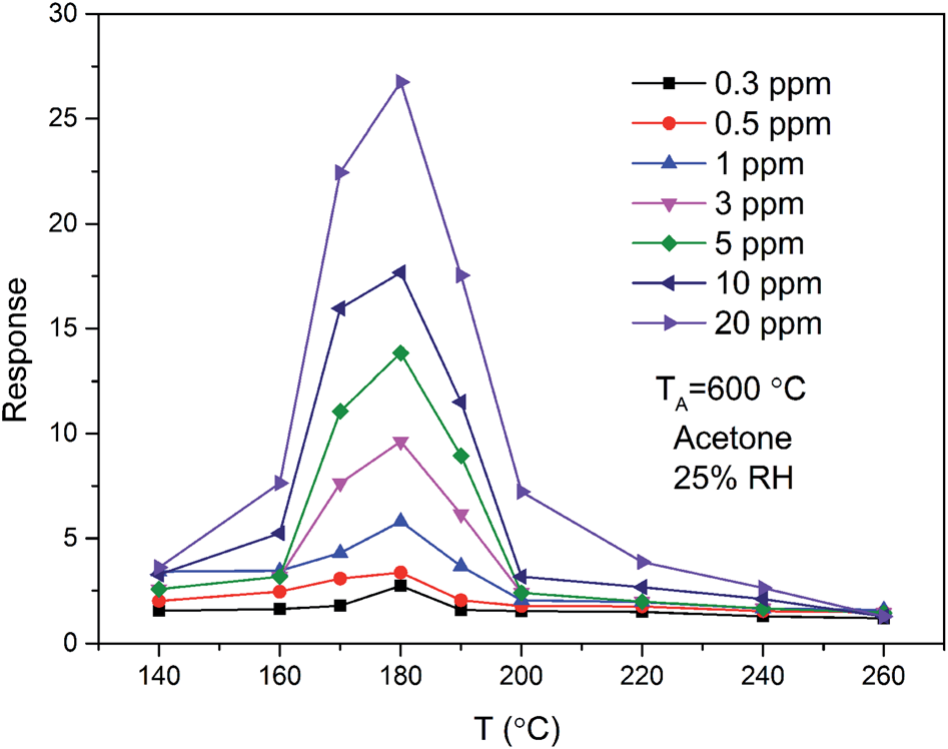
The temperature dependence of response to different concentrations of acetone gas for ZnO/SnO_2_ annealed at 600 °C.

The magnitude of the response as a function of temperature at different exposure levels of acetone for ZnO/SnO_2_ sensor is shown in Fig. 6. To acetone of different concentrations, all the response curves of the samples exhibited a trend of increase-maximum-decrease tendency. The response of the ZnO/SnO_2_ sensor annealed at 600 °C increases with increasing of the acetone concentration and attains the maximum at 180 °C. The peak of the gas-sensing sensitivity curve may be caused by the chemical absorption.^18^

**Fig. 7:**
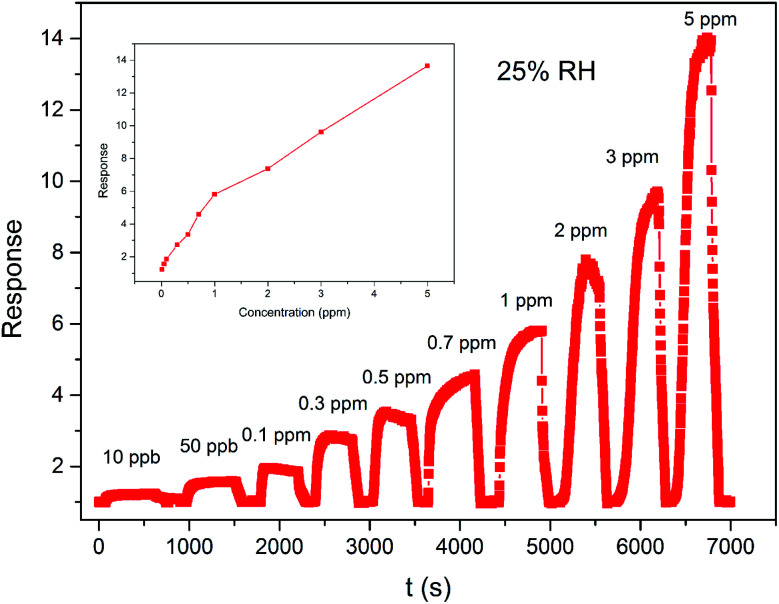
Response and recovery characteristic curve of the sensor based on ZnO/SnO_2_ annealed at 600 °C to different concentrations of acetone (the inset shows response *versus* acetone concentration curve of the gas sensor).

Fig. 7 present the dynamic response–recovery curvy of the ZnO/SnO_2_ sensors as a function of the acetone concentration. It can be clearly seen that the sensor signal shows an immediate response to change in the acetone concentration. After several repeated cycles between the acetone gas and fresh air, the response of the sensor can still recover to the initial state, indicating that the sensor has good reversibility. With the acetone concentration increase from 0.01 to 5 ppm, the response increases. The response/recovery times were about 57 s and 63 s to 0.3 ppm acetone, respectively. The inset of Fig. 7 shows the response of ZnO/SnO_2_-sensor *vs.* acetone concentration at 180 °C and 25% RH. It can be seen from Fig. 7 that sub-ppm scale acetone gas can be detected using ZnO/SnO_2_-sensor and the response is about 1.23 to 0.01 ppm acetone. Table 1^19–28^ shows a comparison of acetone sensing of ZnO/SnO_2_ sensor in the current work with those of some ZnO, SnO_2_-based acetone sensors reported in the literature.

**Table 1:** Acetone sensing properties for ZnO and SnO_2_ based semiconductor sensors

Materials	Preparation method	Response	Concentration	*T*_O_	*T*_A_	Ref.
SnO_2_ nanowires	Hydrothermal approach	6.8	20 ppm	290 °C	600 °C	19
SnO_2_ hollow microspheres	Hydrothermal method	16	50 ppm	200 °C	500 °C	20
SnO_2_ thin films	Dip-coating	19	8 ppm	Room temperature	500 °C	21
SnO_2_ nanobelts	Electrospinning method	6.7	5 ppm	260 °C	600 °C	22
SnO_2_–ZnO hetero-nanofibers	Electrospinning	85	100 ppm	300 °C	600 °C	13
SnO_2_–TiO_2_	Sol–gel method	55	200 ppm	340 °C	450 °C	23
ZnO thin film	Spray pyrolysis	1.42	1000 ppm	320 °C	—	24
ZnO hollow nanofibers	Electrospinning	7.1	1 ppm	220 °C	600 °C	25
ZnO particles	Co-sputtering	10	500 ppm	400 °C	600 °C	26
ZnO thin films	Sol–gel	8.11	100 ppm	200 °C	550 °C	27
Dumbbell-like ZnO	Solution method	16	50 ppm	300 °C	—	28
ZnO/SnO_2_	Sol–gel	3.36	0.5 ppm	180 °C	600 °C	Present work

**Fig. 8:**
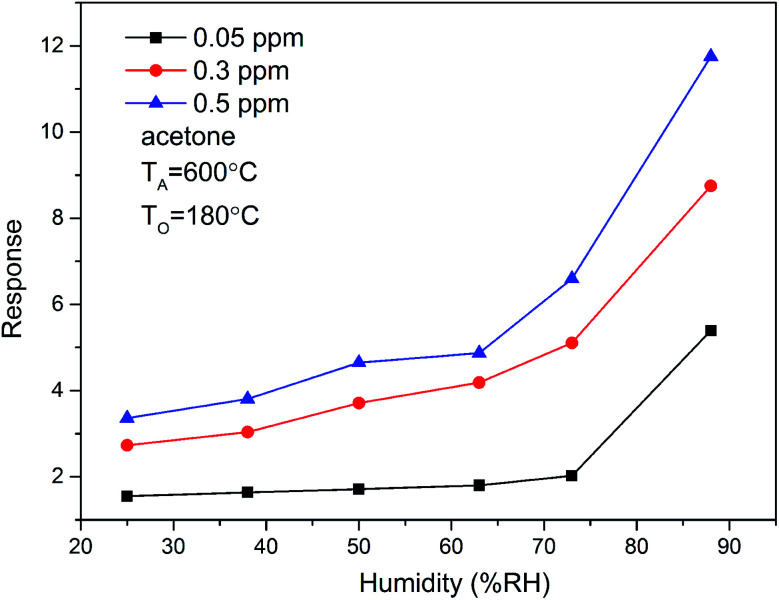
The relative humidity dependence of the response of ZnO/SnO_2_ (with *T*_A_ = 600 °C) for acetone at 180 °C.

Water is omnipresent and has vital effect on the sensing performance of metal oxide semiconductors, ensures continuous interest of surface scientists in the interaction of water with inorganic materials.^29–34^ In the present study, we also investigated the effect relative humidity (RH) on the sensing performance. Fig. 8 shows the response of ZnO/SnO_2_ sensor to acetone gas under different relative humidity. The results show that the response of the sensor to acetone gas increases with the increase of relative humidity. Thus, humidity substantially enhanced the response of ZnO/SnO_2_ to acetone.

**Fig. 9:**
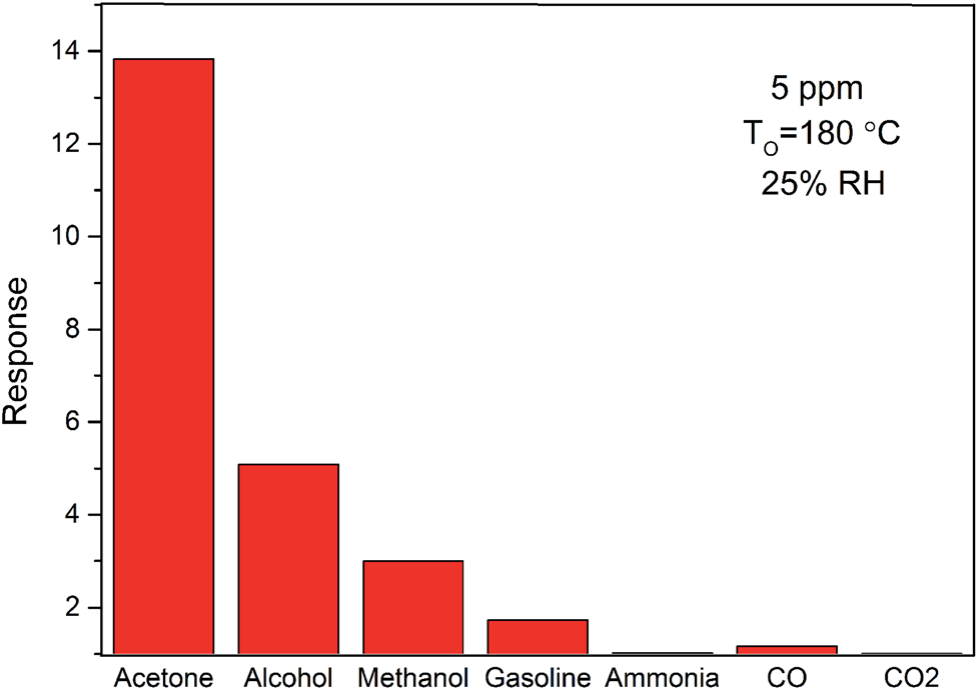
The sensitivities of sensors based on ZnO/SnO_2_ to 5 ppm different gases at an operating temperature of 180 °C in the background of ambient air (with the room temperature humidity of 25% RH).

Selectivity is another important parameter used to evaluate the sensing performance of gas sensors. To understand the selective behavior of ZnO/SnO_2_ at optimal operating temperature (180 °C), the selectivity for 5 ppm different reducing gases is shown in Fig. 9. The response values are about 13.83, 5.09, 2.99, 1.73, 1.02, 1.17 and 1.002 for acetone, alcohol, methanol, gasoline, ammonia, CO and CO_2_, respectively. The response of the ZnO/SnO_2_-based sensor to acetone was more than two times higher than other gases, indicating a substantial selectivity. The higher sensitivity of ZnO/SnO_2_ to acetone than to other gases maybe due to the aldehyde group in acetone. Thus, to reduce the influence of OH^−^ and improve the selectivity, we'd better used sensors based ZnO/SnO_2_ in a dry environment. What's more, when acetone and other reducing gases containing carbonyl or hydroxyl group co-exist in the atmosphere, an array of thick film sensors including ZnO/SnO_2_ and SnO_2_ thick-film elements can be used to distinguish these gases.

**Fig. 10:**
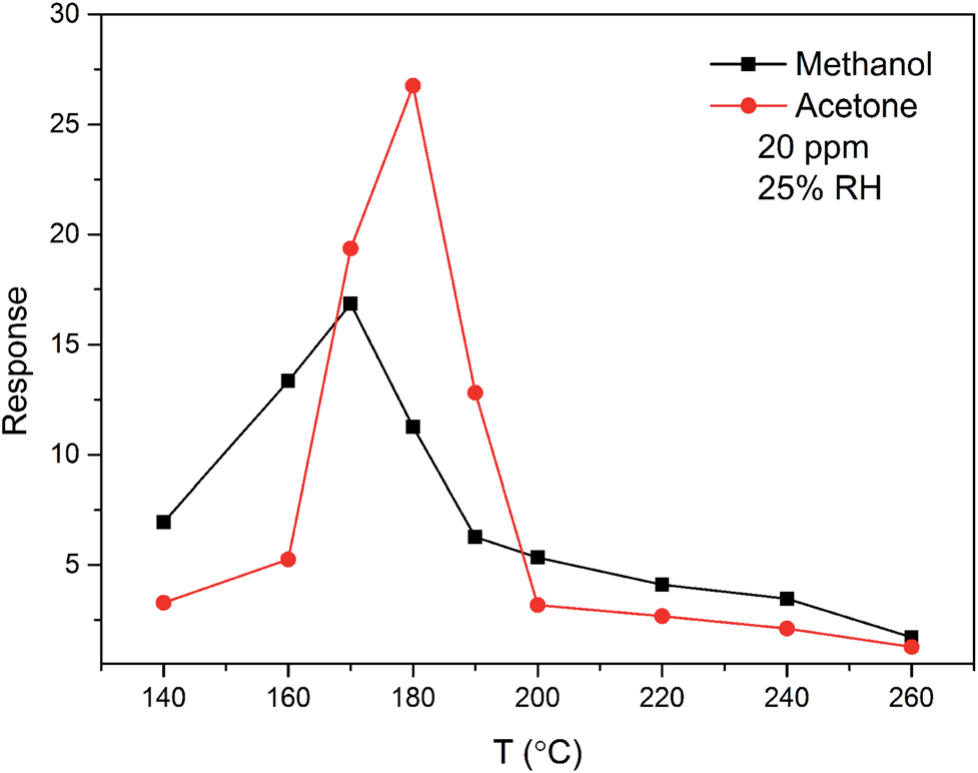
Temperature dependence of the sensitivity to 20 ppm acetone and methanol gas for ZnO/SnO_2_ (with *T*_A_ = 600 °C).

**Fig. 11:**
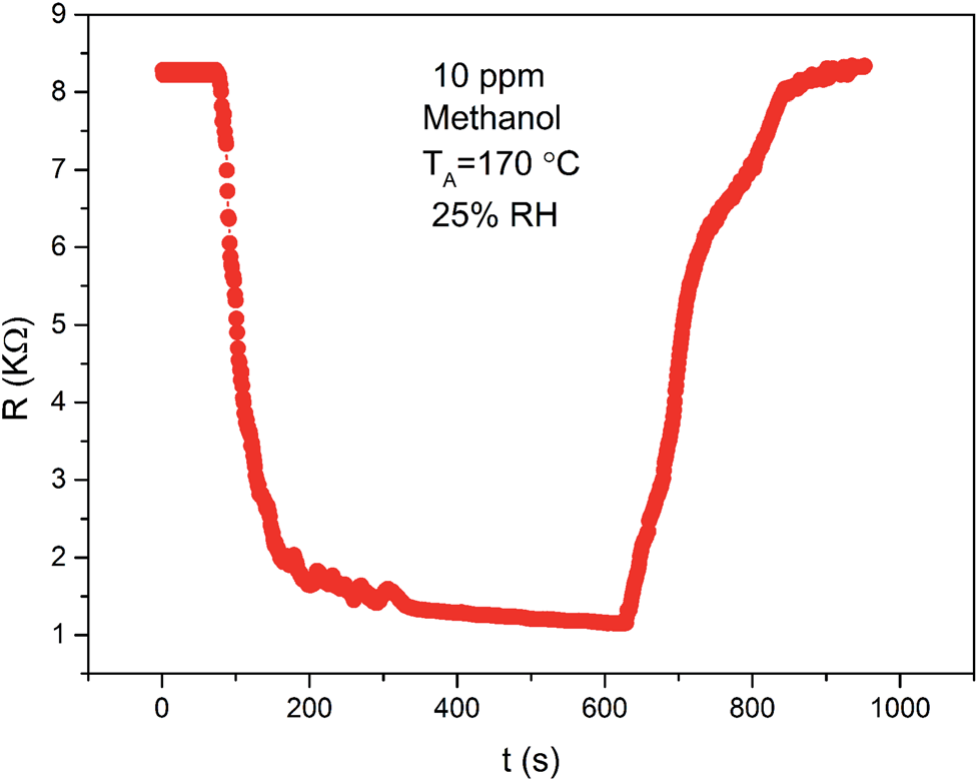
Transient resistance characteristic of ZnO/SnO_2_ (with *T*_A_ = 600 °C) exposed to 20 ppm methanol vapour at 170 °C.

The sensing property of ZnO/SnO_2_ for methanol is seldom reported. To compare the gas sensing property of the material to acetone and methanol, the response was examined as a function of temperature for 20 ppm acetone and methanol. From Fig. 10, it is seen that the ZnO/SnO_2_ possesses different optimum working temperatures for detecting of acetone and methanol. The maximum sensitivity (26.75) to acetone is observed while operating at 180 °C and the maximum sensitivity to methanol is 16.87 at 170 °C. Resistance transients of ZnO/SnO_2_ to 10 ppm methanol is shown in Fig. 11. It can be seen that the present ZnO/SnO_2_ sensor shows poor response–recovery property to methanol than to the acetone gas.

**Fig. 12:**
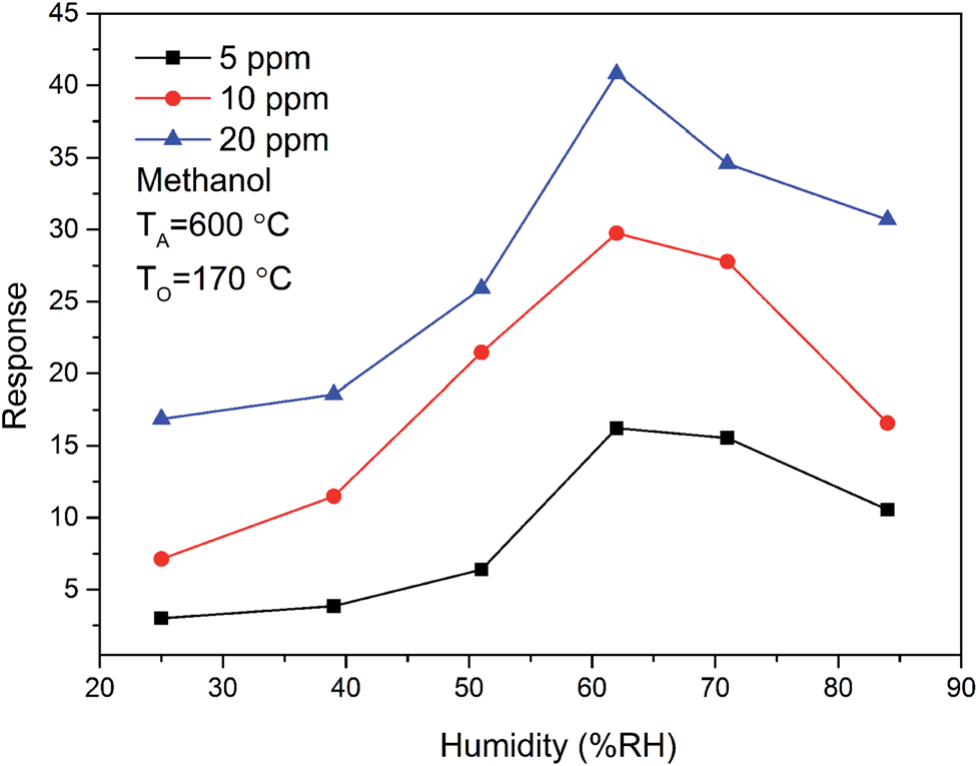
The relative humidity dependence of the response of ZnO/SnO_2_ (with *T*_A_ = 600 °C) for methanol at 170 °C.

In addition, the methanol sensing performance of ZnO/SnO_2_ sensor at different relative humidity (RH) was also studied. The gas sensing response of ZnO/SnO_2_ sensor to 5, 10 and 20 ppm methanol at 170 °C at different relative humidity is shown in Fig. 12. When the relative humidity of the test environment is 60%, the response of methanol increased up to the maximum value. Then the response value was decreased with the increase of relative humidity. The measured maximum response of ZnO/SnO_2_ sensor is about 26.22, 29.75 and 40.80 to 5, 10 and 20 ppm methanol at 60% RH, respectively.

The gas sensing mechanism of metal oxide semiconductors-based sensor could be attributed to the change in electrical conductivity lies in the adsorption and desorption of chemisorbed oxygen species on the surface.^6,35,36^ When the ZnO/SnO_2_ sensor is exposed to air, the oxygen (O_2_) molecules are adsorbed on the surface of ZnO/SnO_2_ and ionized to various chemical absorptive states (O_2_^−^, O^−^, O^2−^) by trapping electrons from ZnO/SnO_2_. Then a space charge layer can be formed. When exposed to reducing gas such as acetone and methanol, the oxygen ions adsorbed on the surface of the ZnO/SnO_2_ sensor would interact with acetone to release electrons. The process can be expressed as follows:

1 C_3_H_6_O + 8O_(ads)_^−^ → 3CO_2_ + 3H_2_O + 8e^−^

2 CH_3_OH + 3O_(ads)_^−^ → CO_2_ + 2H_2_O + 3e^−^

During this process, the trapped electrons were released back to the conduction band of ZnO/SnO_2_, so that the resistance decrease. The explanation is in good agreement with the sensing response of ZnO/SnO_2_ to acetone and methanol.

As traditional n-type semiconductors, ZnO and SnO_2_ have been confirmed to have great potential as sensing materials. Compared with primary ZnO and SnO_2_, the improvement of acetone sensing performances of the ZnO/SnO_2_ sensor can be well interpreted by the formation of n–n heterojunctions at the ZnO/SnO_2_ interfaces.^37–40^ ZnO and SnO_2_ have different work function and thus the n–n heterojunction could be generated at the interface of the two semiconductors. The electrons in SnO_2_ will transfer to ZnO until their Fermi levels align. As a result, a wide “accumulation layer” the surface of ZnO was well formed that plays an important role in the sensing reactions. The above process will lead to more oxygen ions formed by capturing free electrons from the accumulation layer on the surface of ZnO. Thus, more acetone and methanol gas will react with these absorbed oxygen ions leading to the strong promotion of sensitivity.

The effect of the presence of water on the sensitivity of acetone and methanol depends on the amount of water vapor. For acetone sensing property, the higher the testing humidity was, the more sensitive of ZnO/SnO_2_ sensor is. This is believed to be due to the hydroxyl species formed at the sensor surface, which facilitates the response for acetone.^3,30^ For methanol gas, when the relative humidity increases from 25% to 84%, the sensor response starts to increase, reaches a peak around 63%, and then decreases. At low relative humidity, with the increase of relative humidity, more hydroxyl species are formed on the sensor surface, and more electrons transfer between H_2_O and the ZnO/SnO_2_ surface, thus the response increases. At high relative humidity, hydroxyl groups absorbed on the surface of the sensor compete with the adsorbed species, resulting in the decrease of adsorbed species,^3^ and this leads to a decrease of the response to methanol gas. The difference in gas sensitivity between detecting acetone and methanol by the ZnO/SnO_2_ sensor under high humidity conditions is due to the different functional groups of acetone and methanol. Even in dry air, the different functional groups may also lead to a higher sensitivity of ZnO/SnO_2_ to acetone than to methanol.

## Conclusions

In the present work, ZnO/SnO_2_ hybrid sensing nanostructures were synthesized by sol–gel method. The structures and morphologies of the obtained products were characterized by several technical methods. The as-prepared ZnO/SnO_2_ nanostructures exhibited enhanced sensing properties, low detection limit (ppb-level) for acetone detection. For example, the sensitivities to 0.01 and 5 ppm acetone are 1.23 and 13.83, respectively. The curve of sensitivity *versus* acetone concentration is almost linear in the concentration range of 0.01–5 ppm. This improvement could be attributed to the formation of heterojunctions between ZnO and SnO_2_. The effect of humidity was also considered, and humidity enhances the response of ZnO/SnO_2_ to acetone. The plausible gas-sensing mechanism was also discussed.

## Conflicts of interest

There are no conflicts to declare.

## Supplementary Material
